# Development of Polymeric-Based Formulation as Potential Smart Colonic Drug Delivery System

**DOI:** 10.3390/polym14173697

**Published:** 2022-09-05

**Authors:** Mohammad F. Bayan, Saeed M. Marji, Mutaz S. Salem, M. Yasmin Begum, Kumarappan Chidambaram, Balakumar Chandrasekaran

**Affiliations:** 1 Faculty of Pharmacy, Philadelphia University, P.O. Box 1, Amman 19392, Jordan; 2 Faculty of Pharmacy, Jordan University of Science and Technology, P.O. Box 3030, Irbid 22110, Jordan; 3 Department of Pharmaceutics, School of Pharmacy, King Khalid University, Abha 61421, Saudi Arabia; 4 Department of Pharmacology, School of Pharmacy, King Khalid University, Abha 62529, Saudi Arabia; 5 Department of Pharmaceutical Chemistry, School of Pharmacy, ITM University, Gwalior 474001, India

**Keywords:** 5-amino salicylic acid, smart delivery system, sustainable polymers, triggered drug delivery, ulcerative colitis

## Abstract

Conventional oral formulations are mainly absorbed in the small intestine. This limits their use in the treatment of some diseases associated with the colon, where the drug has to act topically at the inflammation site. This paved the way for the development of a smart colonic drug delivery system, thereby improving the therapeutic efficacy, reducing the dosing frequency and potential side effects, as well as improving patient acceptance, especially in cases where enemas or other topical preparations may not be effective alone in treating the inflammation. In healthy individuals, it takes an oral medication delivery system about 5 to 6 h to reach the colon. A colonic drug delivery system should delay or prohibit the medication release during these five to six hours while permitting its release afterward. The main aim of this study was to develop a smart drug delivery system based on pH-sensitive polymeric formulations, synthesized by a free-radical bulk polymerization method, using different monomer and crosslinker concentrations. The formulations were loaded with 5-amino salicylic acid as a model drug and Capmul MCM C8 as a bioavailability enhancer. The glass transition temperature (Tg), tensile strength, Young’s modulus, and tensile elongation at break were all measured as a part of the dried films’ characterization. In vitro swelling and release studies were performed to assess the behavior of the produced formulations. The in vitro swelling and release evaluation demonstrated the potential ability of the developed system to retard the drug release at conditions mimicking the stomach and small intestine while triggering its release at conditions mimicking the colon, which indicates its promising applicability as a potential smart colonic drug delivery system.

## 1. Introduction

Orally delivered dosage forms are the most commonly used dosage forms, due to the ease of administration and convenience. The aim of any successful oral drug delivery system is to deliver the therapeutic agent to the site of action with proper dosing and timing [1]. This can be guaranteed through developing a smart delivery system, which elicits a stimulus-responsive drug release. In these systems, an external or internal stimuli, such as pH change, can trigger the drug release. As a consequence, this can reduce the required doses, reduce potential side effects, increase patient compliance, and improve therapeutic efficacy [2]. The development of such a system requires an understanding of the mechanism of action of the drug, site of action, physicochemical properties, residence time, and the environment that the dosage unit will pass through after administration, so the system can be developed to trigger the drug depending on its environment within the body. The pH and residence time vary within the gastrointestinal tract. The pH changes from acidic in the stomach to relatively basic in the small and large intestines [3]. The drug residence time is estimated to be 1–4 h in the stomach, around 4 h in the small intestine, and around 10 h in the large intestine [2]. These changes have paved the way to develop a pH–time-dependent drug delivery system aiming to deliver the drug in the intestine, for example. This can be achieved via employing polymers that retard the drug in the acidic environment while permitting its release at the basic environment, where the polymer swells or dissolves. Increased attention has been given to these systems to treat some diseases, such as inflammatory bowel disease, which can affect specific parts of the large intestine. Conventional oral dosage forms are mainly absorbed in the small intestine, and this limits their use in the treatment of colon disorders, where the topical effect of the medicinal agent is important at the inflammation sites [4]. This advocated the need to fabricate a colonic oral delivery system especially in some severe and specific cases, where the topical dosage form (such as an enema) may not be effective alone in treating the inflammation. This smart delivery can be achieved using a pH–time-dependent system that employs a polymer capable of preventing/retarding the drug release in the upper gastrointestinal tract (stomach and small intestine) while permitting its release in the lower gastrointestinal tract. The importance of developing a smart colonic delivery system is not justified only for the local treatment of colonic disorders, but it is also extended to the systemic delivery of some agents, such as peptides, proteins, and anti-diabetic and anti-hypertensive agents. Crohn’s disease and ulcerative colitis are the main inflammatory bowel disorders, where the first can affect any part of the intestine, while the second is mainly affecting the colon [5]. Persons affected with this disease have to use a lifelong remedy as there is no permanent treatment for this condition. Additionally, the available remedies are not always effective and can cause severe side effects [6]. Ulcerative colitis is usually treated using 5-amino salicylic acid (also called mesalamine), prescribed as oral and topical dosage forms, as a first choice, for the local treatment of the inflamed parts of the large intestine. 5-amino salicylic acid is considered as class IV in the Biopharmaceutical Classification System (this class characterized by low solubility and low permeability) [7]. The majority of oral-marketed 5-amino salicylic acid dosage forms employs a pH-sensitive polymer coating to withstand the drug release in the stomach while permitting it in the intestine, such as in Asacol^®^, Lialda^®^, Apriso^®^, and Claversal^®^, which use the Eudragit^®^ coating that dissolves at the intestinal pH [8]. The employment of a pH-sensitive polymer can improve the oral delivery of the 5-amino salicylic acid through the pH-dependent swelling, which results in a high concentration gradient and rapid release, as well as high mucoadhesivity, and higher absorption. The pH approach alone may fail to achieve a colonic-triggered delivery of the 5-amino salicylic acid. This is attributed to the inter/intra pH variations and the similarity in the pH of the colon and small intestine. This paved the way to employ a combined pH–time-dependent approach to achieve a colonic-specific delivery [9].

Rehman et al. [10] reported the use of polymers, based on long hydrophobic chains, as a promising large intestinal delayed drug delivery system. The carrier was loaded with 5-amino salicylic and Ibuprofen as the model drugs. The developed system demonstrated a relatively higher in vitro drug release in the simulated large intestinal environment compared to the simulated gastric and small intestinal environments. Another delayed drug delivery system was developed by Mirabbasi et al. [11]. This system employed a polyurethane-grafted chitosan nanoparticle loaded with 5-amino salicylic acid. The in vitro release evaluations had shown the ability of the system to retard the drug release, with a less than 60% cumulative release achieved after 8 h and no burst effect. Synthetic monomers are preferred to be used over the natural monomers in synthesizing crosslinked polymers for pharmaceutical purposes because they permit the flexibility and easiness of modifying the structure of the produced polymer as well as the capability to obtain a large-scale production with uniform and reproducible characteristics [12]. Hydroxyethyl methacrylate has been reported as a biocompatible, chemically and thermally stable synthetic monomer. It is the first and most widely used synthetic monomer in biomedical and pharmaceutical applications [13]. Roointan et al. [14] reported the development of a pH-sensitive drug delivery system based on a cationic polymeric carrier, synthesized using hydroxyethyl methacrylate crosslinked with a dimethylaminoethyl methacrylate monomer, as a promising anti-cancer-specific drug delivery system. Polyethylene glycol diacrylate was used as a crosslinker and doxorubicin as a model drug. This system was developed to trigger the drug release at the acidic pH (the cancer site). In vitro evaluations were conducted in different pH medias, simulating the healthy and cancer sites (pH 7.4 and pH 5.5, respectively). A significant higher drug release was obtained at pH 5.5 compared to that at pH 7.4, which encourages their use as a potential anti-cancer-specific delivery system. Zia et al. [15] investigated the use of hydroxyethyl methacrylate in the development of an anionic polymeric carrier for the potential oral delivery of nonsteroidal anti-inflammatory drugs. The in vitro studies demonstrated a significant higher swelling and release in the simulated intestinal fluid compared to the simulated gastric fluid. The main aim of our study was to develop a smart drug delivery system based on pH-sensitive polymeric formulations, synthesized by free-radical bulk polymerization method, using different monomer and crosslinker concentrations. The formulations were loaded with 5-amino salicylic acid as a model drug and Capmul MCM C8 as a bioavailability enhancer. In vitro swelling and release studies were performed for the produced formulations.

## 2. Materials and Methods

### 2.1. Materials

Hydroxyethyl methacrylate (HEMA), Methacrylic acid (MAA), Dimethylaminoethyl methacrylate (DMAEMA), 5-amino salicylic acid, ethylene glycol dimethacrylate (EGDMA), disodium hydrogen phosphate dodecahydrate, azobisisobutyronitrile (AIBN), potassium chloride, sodium chloride, potassium dihydrogen phosphate, BRAND^®^ stopcock grease, sodium dodecyl sulphate, and sodium hydroxide were purchased from Sigma-Aldrich. Capmul^®^ MCM C8 was purchased from ABITEC. Hydrochloric acid (37%) was purchased from Biosolve Chimie. HPLC-grade water was used in all experiments. The purchased materials were used as supplied with no modification.

### 2.2. Methods

#### 2.2.1. Preparation of Smart Polymeric Formulations

A free-radical thermal bulk polymerization method was used to synthesize 18 copolymerized formulations (Table 1), based on HEMA, MAA, and/or DMAEMA monomers, using different concentrations of EGDMA as a crosslinker, and loaded with capmul MCM C8 and 5-amino salicylic acid, as a dissolution enhancer and model drug, respectively. AIBN was used as a thermoinitiator. A 10 g copolymerized film was produced for each formulation. During preparation, the components of each formulation (in ratios as described in Table 1) were blended together in a 30 mL amber glass bottle, at room temperature, with stirring for 45 min. A 20 mL syringe was used to inject the prepared mixture in a premade mold, designed for all formulations, and then transferred to the oven (preheated to 60 °C), where the polymerization process occurred at 60 °C for 18 h. The mold was made using a medical-grade rubbery silicone tubing (0.76 mm internal diameter, 1.65 external diameter, and 0.445 mm wall thickness), two borosilicate glass sheets (215 × 215 × 3 mm), 8 32 mm-foldback clips, and silicon-coated release liner. The silicone coated sheet was spread onto the glass sheets and the borders of the mold were drawn using the silicone tube on one of the glass sheets, where the other one was flipped onto it and the two sheets held together vertically using the foldback clips. At the end of the synthesis process, each film was soaked in HPLC-grade water placed in a storage box covered with aluminum foil. The water was changed daily to rinse the prepared film and remove any unreacted or unwanted species remaining from the polymerization process. A UV–vis spectrophotometer (Spectroscan 80 D, Biotech Engineering Ltd., London, UK) was used to monitor the washing step. A cork borer no. 1 (5 mm) was used to pierce the produced swollen film into uniform small discs, which were then dried in the oven at 60 °C until reaching a constant weight. The drug entrapment efficiency (EE%) of the produced formulations was calculated using the following formula: EE% = Actual content/theoretical content × 100 %. The theoretical content represents the initial drug concertation used during the preparation (5 % *w*/*w*), while the actual content represents the analyzed drug content of each formulation.

#### 2.2.2. Dynamic Mechanical Thermal (DMT) Analysis

The glass transition temperature of the produced formulations was obtained using Q800 DMT analyzer. The discs were analyzed at a range of 35–160 °C, 1 Hz, and a rate of 3 °C/min. The glass transition temperature was defined as the peak of Tan δ curve. Three replicates were carried out. The mean and the standard deviation were calculated. The data were analyzed statistically using a one-way analysis of variance, followed by Tukey’s multiple comparisons test (*n* = 3, *p* < 0.05).

#### 2.2.3. Mechanical Properties

The mechanical properties of the produced polymeric formulations were characterized using a TA-XT plus texture analyzer. The dried films (25 × 10 mm) were clamped between the grips, leaving a constant length of the films below stress (20 mm). The upper clamp was lifted at a constant speed of 0.5 mm/s until fracturing the film. The tensile strength, Young’s modulus, and tensile elongation at break were determined from the stress–strain curve. Three replicates were carried out. The mean and the standard deviation were calculated. The data were analyzed statistically using a one-way analysis of variance, followed by Tukey’s multiple comparisons test (*n* = 3, *p* < 0.05).

#### 2.2.4. In Vitro Swelling Evaluation

The in vitro swelling behavior of the produced polymeric discs was evaluated in a biobase thermostatic shaking water bath SWB-A, at 37 °C in buffers of equal ionic strength, at pH 1.2 and pH 7.4. Three replicates of each formulation as dried discs were initially weighed and placed in amber glass vials. Each vial was then filled with 10 mL buffer, previously kept at 37 °C in the thermostatic shaking bath. The discs were withdrawn from the vials at predetermined time points using forceps and placed on a thick medical tissue, where they were blotted gently before weighing and immersing them back in their vials in the thermostatic shaking bath. The equilibrium swelling ratio and the swelling behavior of the produced formulations were investigated via plotting the swelling ratios obtained at each time point (calculated using Equation (1)) versus the time. The swelling rate of each formulation was investigated via fitting the swelling ratios of the first 7 h to the Korsmeyer–Peppas model. All data were analyzed statistically using a two-way analysis of variance test, followed by Tukey’s multiple comparisons test (*n* = 3, *p* < 0.05). The statistical tests and the graphs were made using a GraphPad Prism software version 9.4.0.

(1) $$Swelling\;ratio\;\left(\%\right)=\left[\frac{The\;weight\;of\;the\;swollen\;disc\;–\;The\;inital\;weight\;of\;the\;dried\;disc}{The\;weight\;of\;the\;swollen\;disc}\right]\times 100\%\;$$

#### 2.2.5. In Vitro Drug Release Studies

The in vitro release of the model drug (5-amino salicylic acid) was investigated, using a modified method of Heelan and Corrigan, in a biobase thermostatic shaking water bath SWB-A, operating at 100 round per minute and 37 °C in buffers of equal ionic strength, at pH 1.2 and pH 7.4 [16]. Three replicates of each formulation were placed in 28 mL McCartney bottles. Each bottle was then filled with 20 mL buffer, previously kept at 37 °C in the thermostatic shaking bath. A 0.5 mL sample was withdrawn at predetermined time points and replaced with 0.5 mL fresh buffer. The 0.45 µm syringe filters were used to filter the withdrawn samples prior measuring their absorbance in the UV–vis spectrophotometer at 300 nm (pH 1.2) and 330 nm (pH 7.4). Fully validated calibration curves were constructed at the two pH values to determine the concentration of the model drug. The release profiles of 5-amino salicylic acid were constructed via plotting the cumulative release percentage achieved at each time point versus the time. The release rate and mechanism of the drug release were examined after fitting the first 60% of the release data to the Korsmeyer–Peppas model. All data were analyzed statistically using a two-way analysis of variance test, followed by Tukey’s multiple comparisons test (*n* = 3, *p* < 0.05). The statistical tests and the graphs were made using a GraphPad Prism software version 9.4.0.

## 3. Results and Discussion

### 3.1. Preparation of Smart Polymeric Formulations

The main objective of this work was to develop a smart oral drug delivery system capable of delivering drugs with poor solubility and/or poor permeability to their site of action, selectively, with proper dosing and timing. To develop this system, a pH–time-dependent approach was designed, based on copolymerized formulations crosslinked by EGDMA and loaded with capmul MCM C8 (dissolution/bioavailability enhancer). The polymeric smart system was based on HEMA, HEMA copolymerized with MAA, or HEMA copolymerized with DMAEMA. The optimization of this smart system was studied in this work to achieve a colon-specific drug delivery, thereby improving the therapeutic efficacy, reducing the dosing frequency and potential side effects, as well as improving patient acceptance. Capmul^®^ MCM products are usually obtained from the esterification of vegetable-sourced acids with glycerin. They have been extensively used in food products, such as confectionery, ice creams, bakery, and chewing gums. They can improve the dissolution/permeability of poorly soluble/absorbed drugs [17]. Shailendrakumar et al. [18] employed a capmul^®^ MCM product with palm oil to improve the oral bioavailability of pentoxifylline. In another work, Bayan et al. [19] reported the potential use of capmul MCM C8 to improve the dissolution of poorly soluble drugs. The produced polymeric formulations (Table 1) were loaded with 5-amino salicylic acid as a model dug, which is characterized by poor solubility/permeability and used as a first-choice drug in the treatment of inflammatory bowel diseases that affect mainly the colon. A free-radical thermal polymerization technique was used to successfully synthesize eighteen polymeric formulations using AIBN as a thermal initiator. The drug-loaded formulations showed a satisfactory drug entrapment efficiency of ~92% (Table 2).

### 3.2. Dynamic Mechanical Thermal (DMT) Analysis

The Tg values of the produced formulations are summarized in Table 3. All formulations had Tg values between 120 and 130 °C. The employment of the drug demonstrated an insignificant effect on the Tg value of the polymer. The used concentration of the bioavailability enhancer, capmul MCM C8, had no significant effect on the Tg value of the polymer as there was no significant difference between D1 and D2, F1 and F2, D7 and D8, as well as F7 and F8. The Tg value was reduced significantly with an increasing concentration of the crosslinker (EGDMA), as demonstrated by D4, D5, and D6 and F4, F5, and F6. This indicates the formation of a more rigid structure upon increasing the crosslinker concentration. Increasing the MAA and DMAEMA concentrations had no significant effect on the Tg value, as demonstrated between D3 and D4, F3 and F4, D8 and D9, as well as F8 and F9.

### 3.3. Mechanical Properties

The mechanical properties (tensile strength, Young’s modulus, and tensile elongation at break) of the produced formulations are summarized in Table 4. The employment of the drug demonstrated an insignificant effect on the mechanical properties of the polymer. The used concentration of the bioavailability enhancer, capmul MCM C8, had no significant effect on the mechanical properties of the polymer as there was no significant difference between D1 and D2, F1 and F2, D7 and D8, as well as F7 and F8 in the values of the tensile strength, Young’s modulus, and tensile elongation at break. The tensile strength and Young’s modulus values were increased significantly, while the tensile elongation-at-break value reduced significantly, with an increasing concentration of the crosslinker (EGDMA), as demonstrated by D4, D5, and D6 and F4, F5, and F6. This indicates the formation of a more rigid structure upon increasing the crosslinker concentration. Increasing the MAA concentrations had reduced the tensile strength and Young’s modulus values, while it increased the tensile elongation-at-break value, as demonstrated between D3 and D4 as well as F3 and F4. Increasing the DMAEMA concentrations had increased the tensile strength and Young’s modulus values, while it reduced the tensile elongation-at-break value, as demonstrated between D3 and D4 as well as F3 and F4.

### 3.4. In Vitro Swelling Evaluation

**Figure 1:**
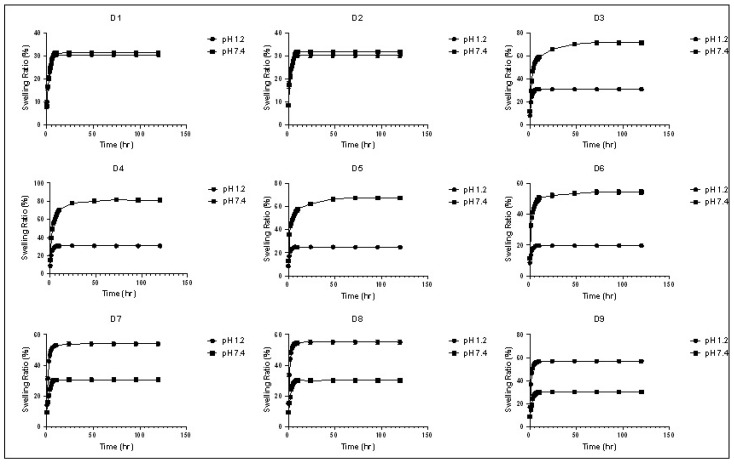
The swelling profiles of the polymeric formulations (mean ± SD, *n* = 3) at pH 1.2 and pH 7.4.

**Figure 2:**
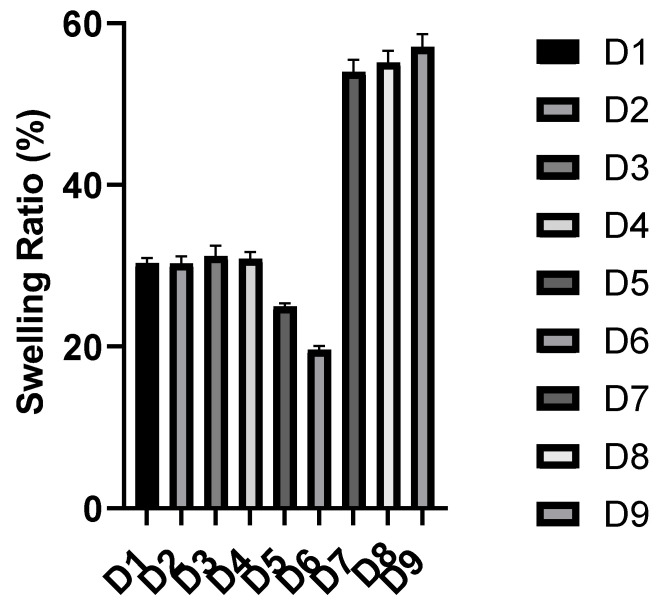
The equilibrium swelling ratios of the polymeric formulations (mean ± SD, *n* = 3) at pH 1.2.

**Figure 3:**
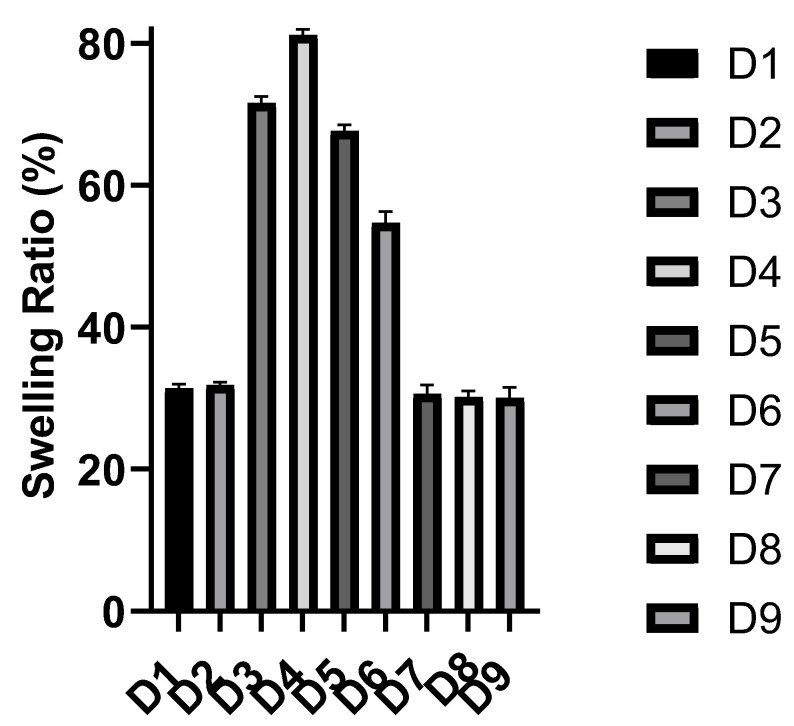
The equilibrium swelling ratios of the polymeric formulations (mean ± SD, *n* = 3) at pH 7.4.

The swelling behavior of a polymer in a fluid is an intrinsic property of the polymeric structure, which occurs as a result of the penetration of the liquid into the voids between the polymeric chains. This behavior is governed by the polymer–fluid and polymer–polymer interactions [20]. The maximum or equilibrium swelling ratio is reached when a balance takes place between these interactions, and it can be affected by some triggers, such as ionic strength, temperature, and pH. The swelling behavior of a polymeric carrier plays an important role in controlling the drug release as it can facilitate the diffusion of the drug through the polymeric matrix as well as the erosion of the polymer [2]. The in vitro swelling behavior of the synthesized polymers was investigated at pH 1.2 and pH 7.4, simulating the pH conditions the drug will face during its way to the colon. The in vitro swelling profile and equilibrium swelling ratio of the synthesized polymers at each pH are presented in Figure 1, Figure 2 and Figure 3 and Supplementary Materials (Figures S1–S11). Table 5 shows the swelling rate constants (k) at each pH, estimated after fitting to the Korsmeyer–Peppas equation. The used concentration of the bioavailability enhancer, capmul MCM C8, had no significant effect on the swelling behavior of the polymers, as there was no significant difference between D1 and D2 as well as D7 and D8 at each pH. The swelling ratio and rate were reduced significantly with an increasing concentration of the crosslinker (EGDMA), as demonstrated by the significant differences in the swelling behavior between D4, D5, and D6 at each pH. This can be attributed to the reduced polymer’s elasticity and the formation of a more rigid structure upon increasing the crosslinker concentration [21]. Polymers, based on HEMA without MAA or DMAEMA, displayed statistically similar swelling profiles at the two pH values, as demonstrated for D1 and D2 at the two pH values. This can be contributed to the absence of ionizable pendant groups in these polymers, so they exist in the neutral form at the simulated gastric and intestinal fluids. Polymers, based on HEMA-co-MAA and HEMA-co-DMAEMA, displayed a pH-responsive swelling. Regarding the HEMA-co-MAA formulations, a significant higher swelling profile was observed at pH 7.4 compared to that at pH 1.2. This can be contributed to the greater ionization of the anionic pendant groups (MAA) in these polymers at the simulated intestinal fluid compared to that at the simulated gastric fluid, so greater electrostatic repulsions between the pendant groups and enhanced swelling [22]. Regarding the HEMA-co-DMAEMA formulations, a significant higher swelling profile was observed at pH 1.2 compared to that at pH 7.4. This can be contributed to the greater ionization of the cationic pendant group (DMAEMA) in these polymers at the simulated gastric fluid compared to that at the simulated intestinal fluid [14]. Increasing the DMAEMA concentration had no significant effect on the swelling profile at the two pH values, as demonstrated between D8 and D9. Increasing the MAA concentration had no significant effect on the swelling profile pH 1.2, while it increased the swelling significantly at pH 7.4, as demonstrated between D3 and D4. The equilibrium swelling ratios of the polymers, based on HEMA and HEMA-co-DMAEMA, were reached within 24 *h* at the two pH values. The equilibrium swelling ratios of polymers, based on HEMA-co-MAA, were reached within 24 h at pH 1.2 and 72 *h* at pH 7.4. equilibrium ratios of ~30, ~25, ~19, and ~55% were obtained for D1–D4, D5, D6, and D7–D9, respectively, at pH 1.2. At pH 7.4, equilibrium ratios of ~30% were obtained for polymers, based on HEMA and HEMA-co-DMAEMA. The HEMA-co-MAA-based polymers exhibited a pH-responsive swelling at pH 7.4 with ~70, ~80, ~67, and ~54% equilibrium ratios obtained for D3, D4, D5, and D6, respectively.

### 3.5. In Vitro Drug Release Studies

**Figure 4:**
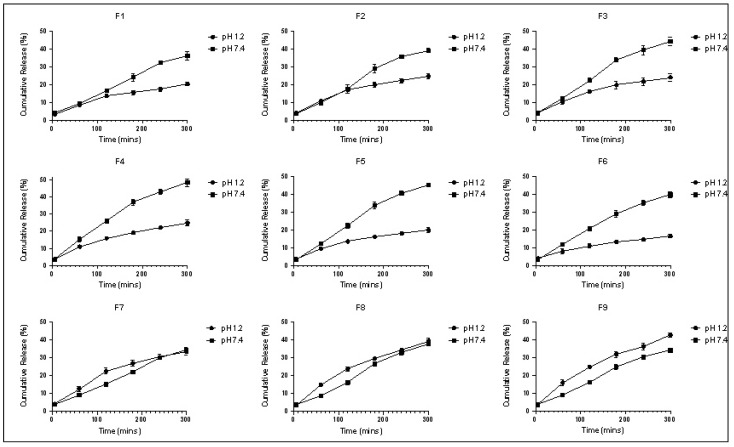
The release profiles of the polymeric formulations (mean ± SD, *n* = 3) at pH 1.2 and pH 7.4.

The in vitro release of the 5-amino salicylic acid from the prepared polymeric formulations was investigated at pH 1.2 and pH 7.4, simulating the environmental conditions that it will face the formulation during its transit through the GI to the colon. The calibration curve of 5-amino salicylic acid at each pH is presented in the Supplementary Materials (Figures S12 and S13). The in vitro release profiles of the 5-amino salicylic acid from the synthesized polymers at each pH are shown in Figure 4 and Supplementary Materials (Figures S14–S24). Table 6 displays the R^2^, *n* values, and release rate constants (*k*) at each pH, estimated after fitting the release data to the Korsmeyer–Peppas model. The used concentration of the bioavailability enhancer, capmul MCM C8, has significantly increased the release profile of the 5-amino salicylic acid, as demonstrated by D1, D2 and D7, D8 at each pH. This can be contributed to forming micelles within the polymeric matrix by the capmul^®^ MCM C8, thus enhancing the dissolution and release of the 5-amino salicylic acid [19]. The release profile of the model drug was also decreased significantly with an increasing concentration of the crosslinker (EGDMA), as demonstrated by F4, F5, and F6 at each pH. This agrees with the in vitro swelling finding, and it is contributed to the reduced polymer’s elasticity and the formation of a more rigid structure [21]. At pH 1.2, the F1–F6 polymeric formulations (based on HEMA and HEMA-co-MAA) demonstrated a higher ability to retard the release of the model drug compared with the HEMA-co-DMAEMA polymeric formulations (F7–F9). After 5 h, a less than ~25% cumulative release was achieved for F1–F6, while a more than ~35% cumulative release was reached for F7–F9 at pH 1.2. This can be justified by the existence of the HEMA- and HEMA-co-MAA-based polymers in a neutral form in the simulated gastric fluid, while the existence of the HEMA-co-DMAEMA-based polymers in the ionized form is due to the ionization of the cationic pendant groups (DMAEMA). This results in greater electrostatic repulsions between the pendant groups (DMAEMA) and exhibits a pH-responsive swelling and release. At pH 7.4, the HEMA-co-MAA-based formulations (F3–F6) demonstrated a higher release rate compared with the other formulations. This can be explained by the presence of these formulations in the ionized form, due to the ionization of the anionic pendant groups (MAA), thus eliciting a pH-responsive swelling and release. F4 achieved the highest cumulative drug release at pH 7.4 with a ~75% cumulative release achieved after 12 h. This formulation also achieved a ~25% cumulative release after 5 h at pH 1.2. An oral drug delivery system is estimated to reach the colon after 5–6 h of administration in healthy persons. A colonic drug delivery system should prevent/retard the drug release during these 5–6 h while permitting its release afterward. The in vitro release studies demonstrated the potential ability of F4 to retard the release of the 5-amino salicylic acid during its residence in the stomach and small intestine while triggering the payload to the colon. This indicates their potential applicability as a smart colonic delivery system. The mechanism of the drug release from a polymeric carrier is usually controlled through diffusion, swelling, and/or chemical cleavage [23]. The first 60% of the release data at the two pH values were fitted to the Korsmeyer–Peppas model.

(2) $$F=k\;t^{n}$$

This exponential model (Equation (2): *F* represents the fraction of drug released at a specific time, *k* represents the release rate constant, *t* is time in hours, and *n* is the release exponent) is widely used with polymeric formulations to investigate *k* and the mechanism of the drug release. Plotting log *F* versus *t* will result in a linear relationship, where the *n* value represents the slope of the line, and its value indicates the mechanism of the drug release. The antilog of the y intercept represents *k*. The mechanism of the drug release is interpreted as a Fickian diffusion if n is less than or equal to 0.5, interpreted as an anomalous release if n is less than 1.0 and greater than 0.5, interpreted as a case 2 transport if n equals 1, and interpreted as a super case 2 transport if n is greater than 1 [24]. As described in Table 6, all the polymeric formulations at pH 7.4 and F7–F9 at pH 1.2 had an *n* value greater than 0.5 and less than 1, which refers to an anomalous mechanism of the drug release. This means that the release of the 5-amino salicylic acid in these formulations is controlled by the diffusion and swelling of the polymer. Other formulations (F1–F6) at pH 1.2 had an *n* value less than 0.5, which refers to a Fickian diffusion mechanism.

## 4. Conclusions

This research work has investigated the development of a smart drug delivery system capable of achieving a colonic-specific drug delivery. A free-radical polymerization method was used to successfully synthesize polymeric formulations loaded with 5-amino salicylic acid as a model drug. The drug-loaded formulations showed a satisfactory drug entrapment efficiency of ~92%. These formulations were based on copolymerized formulations (HEMA, HEMA-co-MAA, or HEMA-co-DMAEMA) crosslinked by EGDMA and loaded with capmul MCM C8 (dissolution/bioavailability enhancer). The optimization of this smart system was investigated in this work to achieve a colon-specific drug delivery, thereby improving the therapeutic efficacy, reducing the dosing frequency and potential side effects, as well as improving patient acceptance. Polymers, based on HEMA-co-MAA, exhibited a significant higher swelling profile at pH 7.4 compared to that at pH 1.2, which is contributed to the greater ionization of the anionic pendant groups (MAA) in these polymers. The in vitro release studies of F4 have also demonstrated the potential ability of this carrier to delay the release of 5-amino salicylic acid during its stay in the stomach and small intestine while triggering the payload to the colon. This makes it promising to achieve a colonic-specific delivery for the potential treatment of colon-associated diseases, such as inflammatory bowel diseases, and to improve the bioavailability of BSC class IV drugs. Further work will assess the in vivo behavior, biocompatibility, and safety of this system.

## Figures and Tables

**Table 1:** The compositions of the synthesized copolymerized formulations.

Formula	HEMA (% *w*/*w*)	MAA (% *w*/*w*)	DMAEMA (% *w*/*w*)	EGDMA (% *w*/*w*)	Capmul MCM C8 (% *w*/*w*)	AIBN (% *w*/*w*)	5-Amino Salicylic Acid (% *w*/*w*)
D1	98	-	-	1	-	1	-
D2	78	-	-	1	20	1	-
D3	68	10	-	1	20	1	-
D4	58	20	-	1	20	1	-
D5	54	20	-	5	20	1	-
D6	49	20	-	10	20	1	-
D7	88	-	10	1	-	1	-
D8	68	-	10	1	20	1	-
D9	58	-	20	1	20	1	-
F1	93	-	-	1	-	1	5
F2	73	-	-	1	20	1	5
F3	63	10	-	1	20	1	5
F4	53	20	-	1	20	1	5
F5	49	20	-	5	20	1	5
F6	44	20	-	10	20	1	5
F7	83	-	10	1	-	1	5
F8	63	-	10	1	20	1	5
F9	53	-	20	1	20	1	5

**Table 2:** The EE% values of the produced formulations.

Formulation	EE%
F1	90.11 ± 1.84
F2	92.73 ± 2.45
F3	92.79 ± 1.97
F4	93.79 ± 1.16
F5	92.48 ± 2.01
F6	91.92 ± 3.07
F7	91.05 ± 0.53
F8	92.31 ± 1.42
F9	92.20 ± 1.38

**Table 3:** The Tg values of the produced polymeric films.

Formulation	Tg (°C)
D1	127.4 ± 1.27
D2	126.6 ± 0.66
D3	123.4 ± 0.95
D4	120.3 ± 0.78
D5	122.6 ± 0.81
D6	125.1 ± 1.15
D7	129.5 ± 1.04
D8	128.1 ± 1.18
D9	128.4 ± 0.71
F1	126.3 ± 0.91
F2	126.0 ± 1.10
F3	122.9 ± 0.87
F4	121.8 ± 1.31
F5	123.0 ± 1.17
F6	125.4 ± 0.57
F7	129.2 ± 1.39
F8	128.6 ± 1.22
F9	129.8 ± 0.62

**Table 4:** The mechanical properties of the produced polymeric films.

Formulation	Tensile Strength (MPa)	Young’s Modulus (MPa)	Tensile Elongation at Break (%)
D1	5.34 ± 0.52	33.02 ± 0.87	2.38 ± 0.19
D2	5.06 ± 0.19	31.07 ± 0.49	2.08 ± 0.29
D3	4.67 ± 0.43	28.47 ± 0.79	2.62 ± 0.12
D4	4.40 ± 0.24	23.99 ± 1.26	3.97 ± 0.32
D5	5.05 ± 0.20	27.42 ± 0.71	3.25 ± 0.21
D6	5.45 ± 0.30	33.76 ± 0.36	2.35 ± 0.25
D7	5.79 ± 0.22	36.64 ± 1.76	2.07 ± 0.58
D8	5.30 ± 0.41	33.21 ± 0.77	2.52 ± 0.31
D9	6.10 ± 0.32	37.79 ± 2.60	2.15 ± 0.60
F1	5.10 ± 0.45	32.00 ± 0.50	2.38 ± 0.38
F2	5.89 ± 1.05	30.99 ± 0.79	1.92 ± 0.10
F3	5.57 ± 0.43	26.86 ± 0.90	2.93 ± 0.50
F4	4.66 ± 0.41	25.09 ± 0.64	3.87 ± 0.23
F5	4.94 ± 0.29	27.60 ± 1.04	3.18 ± 0.27
F6	5.37 ± 0.17	31.61 ± 1.28	2.43 ± 0.29
F7	5.84 ± 0.34	36.48 ± 1.95	2.15 ± 0.47
F8	5.65 ± 0.41	33.98 ± 1.35	2.60 ± 0.23
F9	6.08 ± 0.07	37.06 ± 3.00	2.07 ± 0.50

**Table 5:** The swelling rate constants (*k*) and R^2^ obtained from fitting to the Korsmeyer–Peppas model.

Formulation	R^2^	*k*
D1 pH 1.2	0.987	0.177
D2 pH 1.2	0.999	0.176
D3 pH 1.2	0.982	0.185
D4 pH 1.2	0.979	0.191
D5 pH 1.2	0.980	0.165
D6 pH 1.2	0.994	0.138
D7 pH 1.2	0.982	0.314
D8 pH 1.2	0.982	0.331
D9 pH 1.2	0.979	0.355
D1 pH 7.4	0.997	0.171
D2 pH 7.4	0.998	0.175
D3 pH 7.4	0.995	0.297
D4 pH 7.4	0.985	0.369
D5 pH 7.4	0.969	0.313
D6 pH 7.4	0.965	0.280
D7 pH 7.4	0.985	0.176
D8 pH 7.4	0.973	0.174
D9 pH 7.4	0.971	0.168

**Table 6:** R^2^, *n*, and *k* obtained from fitting the release data to the Korsmeyer–Peppas model.

Formulation	R^2^	*n*	*k*
F1 pH 1.2	0.989	0.444	0.095
F2 pH 1.2	0.994	0.450	0.119
F3 pH 1.2	0.993	0.453	0.115
F4 pH 1.2	0.998	0.449	0.117
F5 pH 1.2	0.997	0.398	0.103
F6 pH 1.2	0.983	0.343	0.090
F7 pH 1.2	0.990	0.518	0.147
F8 pH 1.2	0.994	0.585	0.154
F9 pH 1.2	0.999	0.598	0.162
F1 pH 7.4	0.965	0.556	0.133
F2 pH 7.4	0.969	0.586	0.138
F3 pH 7.4	0.985	0.606	0.159
F4 pH 7.4	0.997	0.634	0.172
F5 pH 7.4	0.989	0.639	0.153
F6 pH 7.4	0.993	0.610	0.142
F7 pH 7.4	0.964	0.541	0.128
F8 pH 7.4	0.961	0.587	0.131
F9 pH 7.4	0.972	0.570	0.127

## Data Availability

The data presented in this study are available on request from the corresponding author.

## Supplementary Materials

The following supporting information can be downloaded at: https://www.mdpi.com/article/10.3390/polym14173697/s1, Figure S1: The swelling profile of the polymeric formulations (mean ± SD, *n* = 3) at pH 1.2; Figure S2: The swelling profile of the polymeric formulations (mean ± SD, *n* = 3) at pH 7.4; Figure S3: The swelling profile of D1 (mean ± SD, *n* = 3) at each pH; Figure S4: The swelling profile of D2 (mean ± SD, *n* = 3) at each pH; Figure S5: The swelling profile of D3 (mean ± SD, *n* = 3) at each pH; Figure S6: The swelling profile of D4 (mean ± SD, *n* = 3) at each pH; Figure S7: The swelling profile of D5 (mean ± SD, *n* = 3) at each pH; Figure S8: The swelling profile of D6 (mean ± SD, *n* = 3) at each pH; Figure S9: The swelling profile of D7 (mean ± SD, *n* = 3) at each pH; Figure S10: The swelling profile of D8 (mean ± SD, *n* = 3) at each pH; Figure S11: The swelling profile of D9 (mean ± SD, *n* = 3) at each pH; Figure S12: Calibration curve of 5-amino salicylic acid at pH 1.2; Figure S13: Calibration curve of 5-amino salicylic acid at pH 7.4; Figure S14: The release profile of the polymeric formulations (mean ± SD, *n* = 3) at pH 1.2.; Figure S15: The release profile of the polymeric formulations (mean ± SD, *n* = 3) at pH 7.4.; Figure S16: The release profile of F1 (mean ± SD, *n* = 3) at each pH; Figure S17: The release profile of F2 (mean ± SD, *n* = 3) at each pH; Figure S18: The release profile of F3 (mean ± SD, *n* = 3) at each pH; Figure S19: The release profile of F4 (mean ± SD, *n* = 3) at each pH; Figure S20: The release profile of F5 (mean ± SD, *n* = 3) at each pH; Figure S21: The release profile of F6 (mean ± SD, *n* = 3) at each pH; Figure S22: The release profile of F7 (mean ± SD, *n* = 3) at each pH; Figure S23: The release profile of F8 (mean ± SD, *n* = 3) at each pH; Figure S24: The release profile of F9 (mean ± SD, *n* = 3) at each pH.
