# Supplementary material for: Development of Polymeric-Based Formulation as Potential Smart Colonic Drug Delivery System
Source: Polymers (Basel). 2022 Sep 5;14(17):3697. doi: 10.3390/polym14173697 (PMC9460644; doi:10.3390/polym14173697)
Supplement: Supplementary file 1 [file polymers-14-03697-s001.zip › polymers-1852641-supplementary.pdf]

Supporting Information

Development of Polymeric-Based Formulation as Potential Smart Colonic Drug Delivery System

Mohammad F. Bayan [1,*], Saeed M. Marji [1], Mutaz S. Salem [1,2], M. Yasmin Begum [3], Kumarappan Chidambaram [4,*] and Balakumar Chandrasekaran [5]

[1]Faculty of Pharmacy, Philadelphia University, P.O. Box 1, Amman 19392, Jordan
[2]Faculty of Pharmacy, Jordan University of Science and Technology, P.O. Box 3030, Irbid 22110, Jordan
[3]Department of Pharmaceutics, School of Pharmacy, King Khalid University, Abha 61421, Saudi Arabia
[4]Department of Pharmacology, School of Pharmacy, King Khalid University, Abha 62529, Saudi Arabia
[5]Department of Pharmaceutical Chemistry, School of Pharmacy, ITM University, Gwalior 474001, India

*Correspondence: mbayan01@qub.ac.uk (M.F.B.); kumarappan@kku.edu.sa (K.C.)

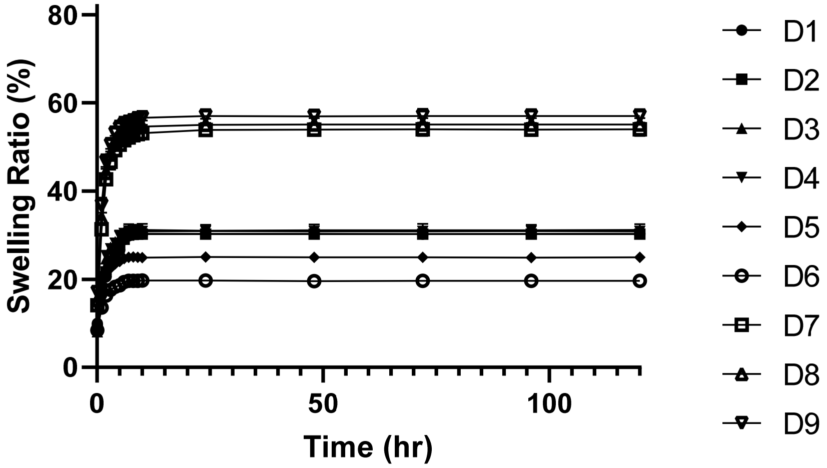

Figure S1: The swelling profile of the polymeric formulations (mean ± SD, n=3) at pH 1.2.

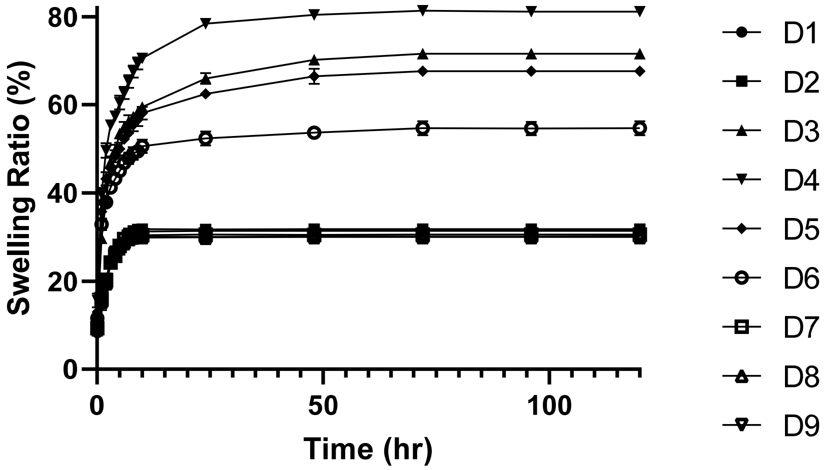

Figure S2: The swelling profile of the polymeric formulations (mean ± SD, n=3) at pH 7.4.

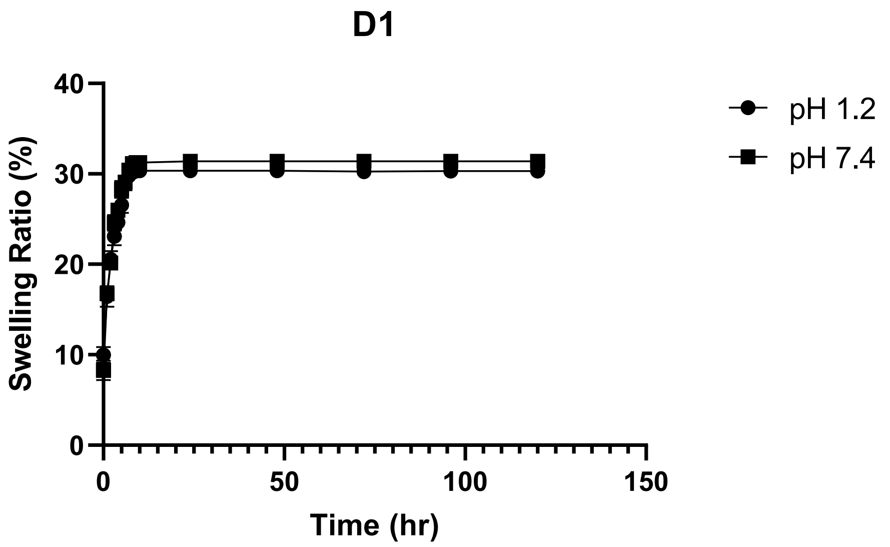

Figure S3: The swelling profile of D1 (mean ± SD, n=3) at each pH.

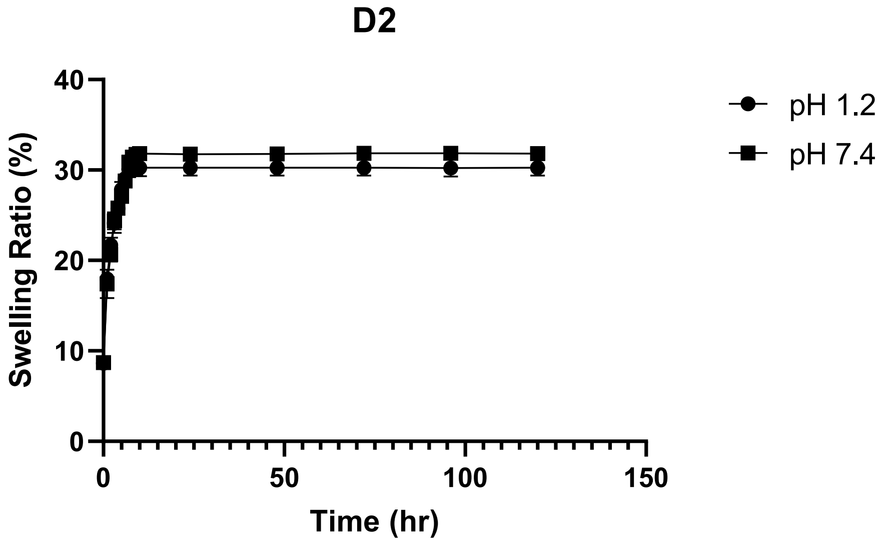

Figure S4: The swelling profile of D2 (mean ± SD, n=3) at each pH.

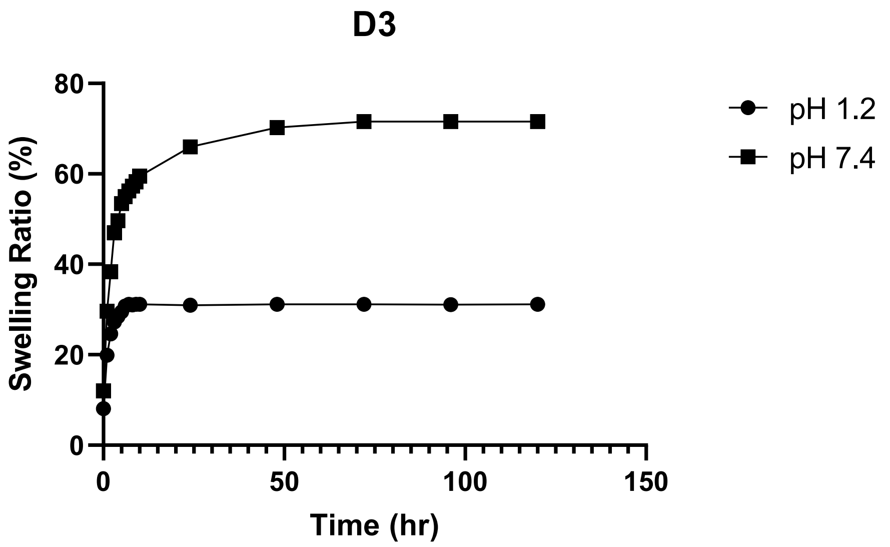

Figure S5: The swelling profile of D3 (mean ± SD, n=3) at each pH.

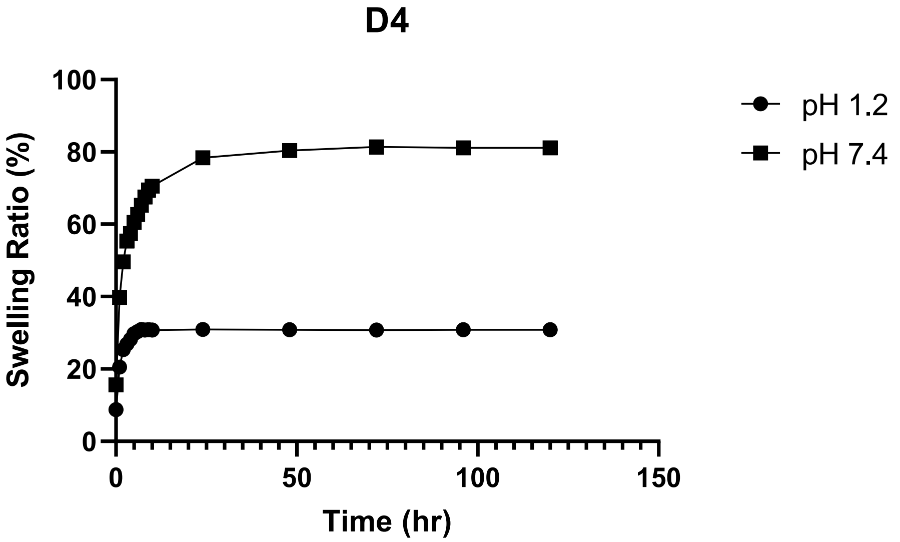

Figure S6: The swelling profile of D4 (mean ± SD, n=3) at each pH.

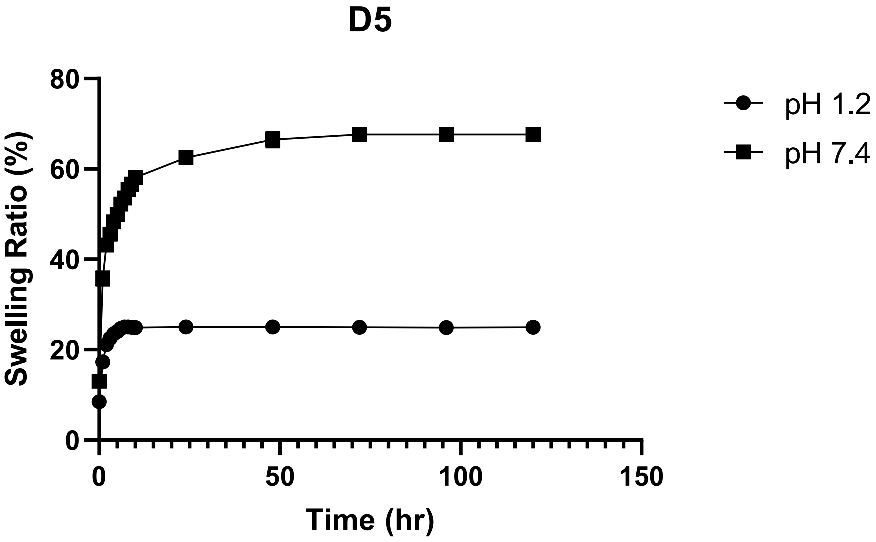

Figure S7: The swelling profile of D5 (mean ± SD, n=3) at each pH.

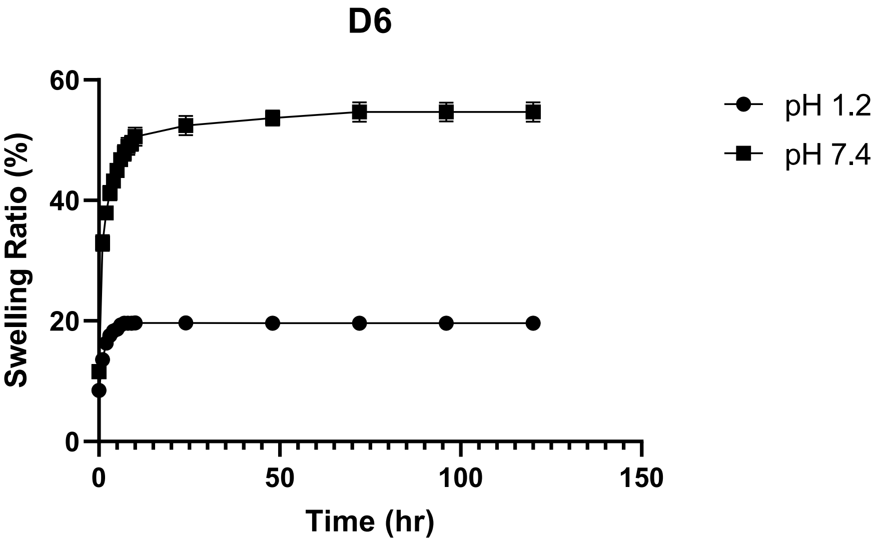

Figure S8: The swelling profile of D6 (mean ± SD, n=3) at each pH.

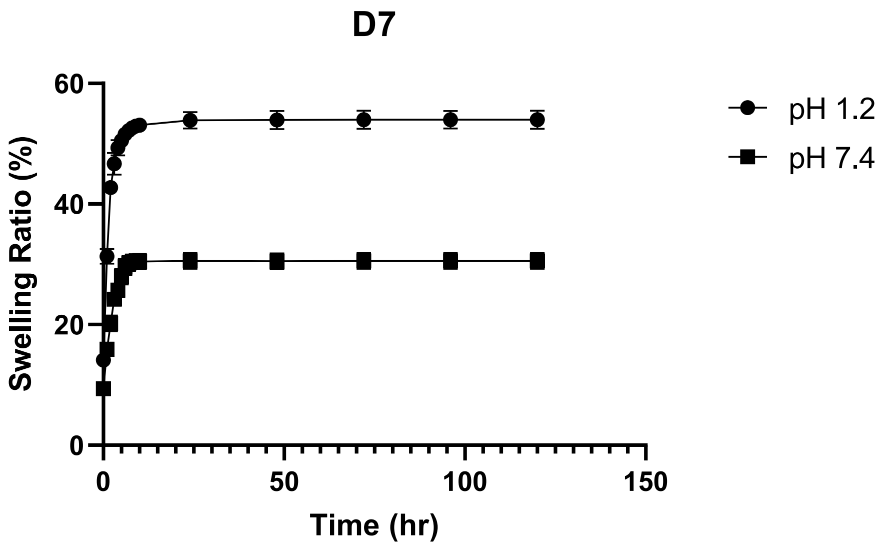

Figure S9: The swelling profile of D7 (mean ± SD, n=3) at each pH.

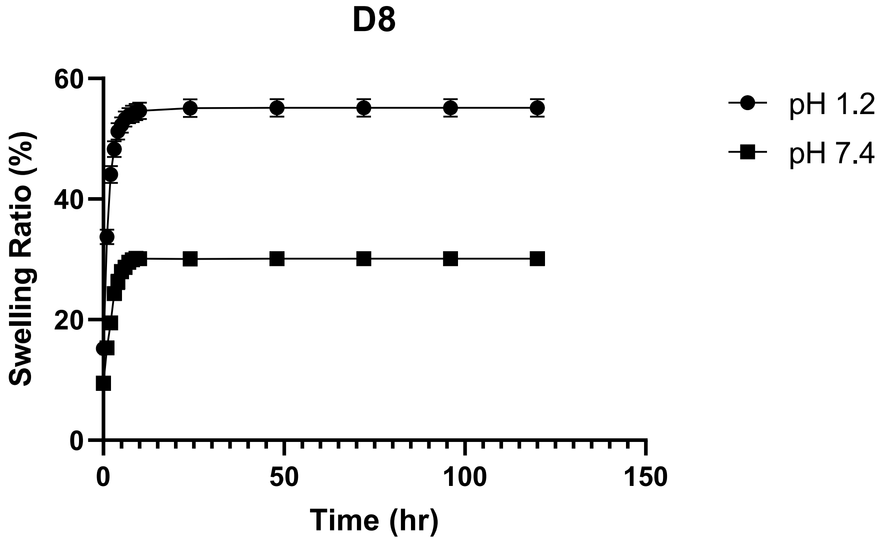

Figure S10: The swelling profile of D8 (mean ± SD, n=3) at each pH.

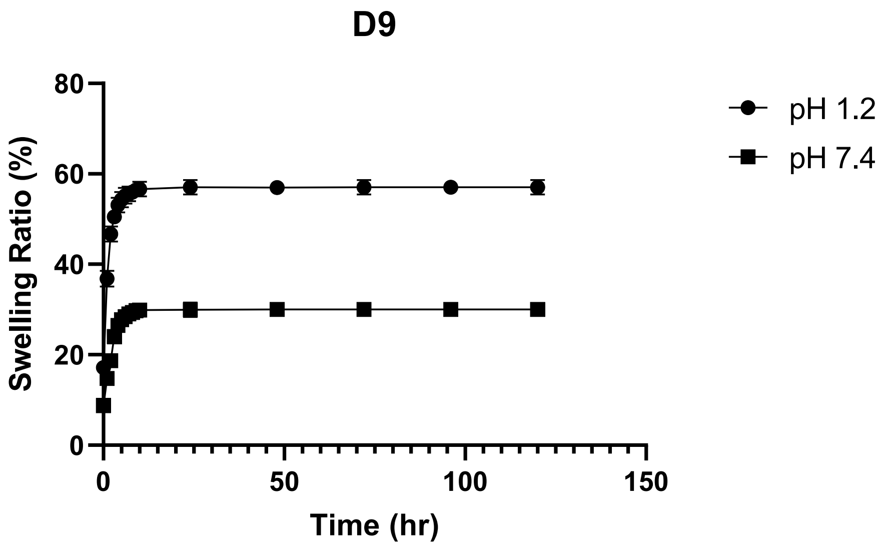

Figure S11: The swelling profile of D9 (mean ± SD, n=3) at each pH.

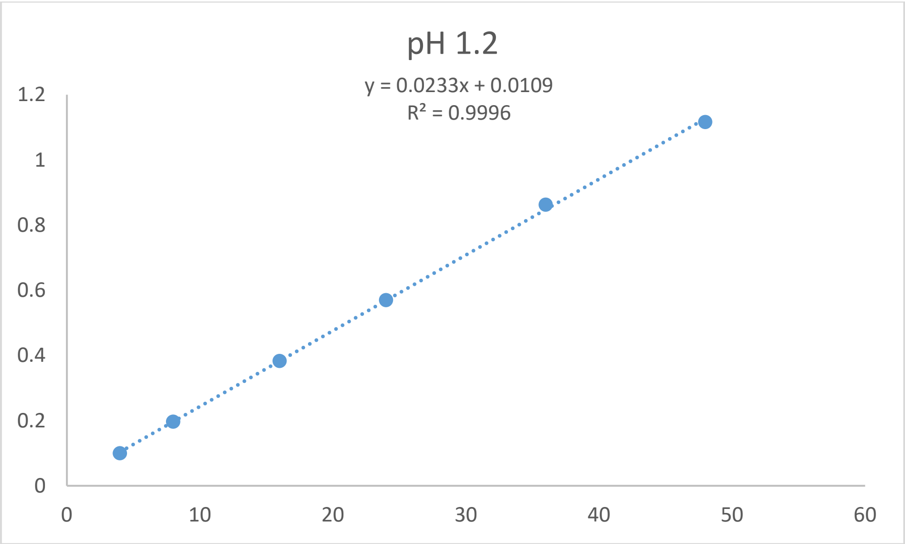

Figure S12: Calibration curve of 5-amino salicylic acid at pH 1.2

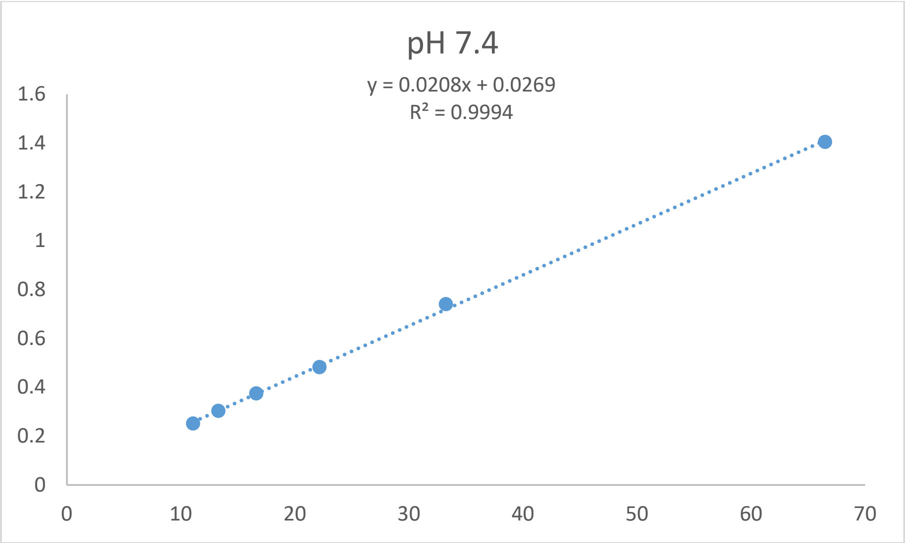

Figure S13: Calibration curve of 5-amino salicylic acid at pH 7.4

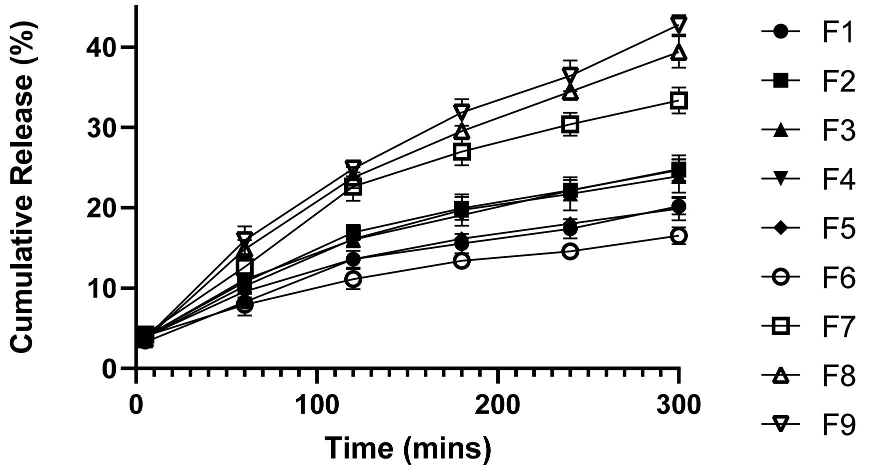

Figure S14: The release profile of the polymeric formulations (mean ± SD, n=3) at pH 1.2.

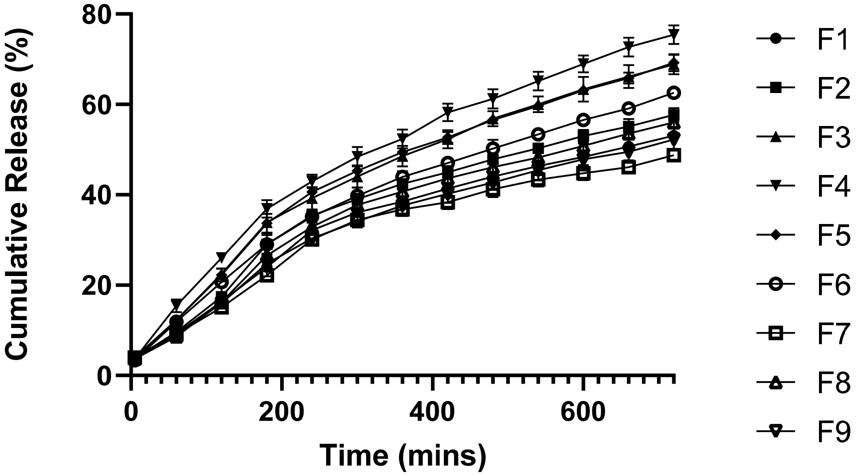

Figure S15: The release profile of the polymeric formulations (mean ± SD, n=3) at pH 7.4.

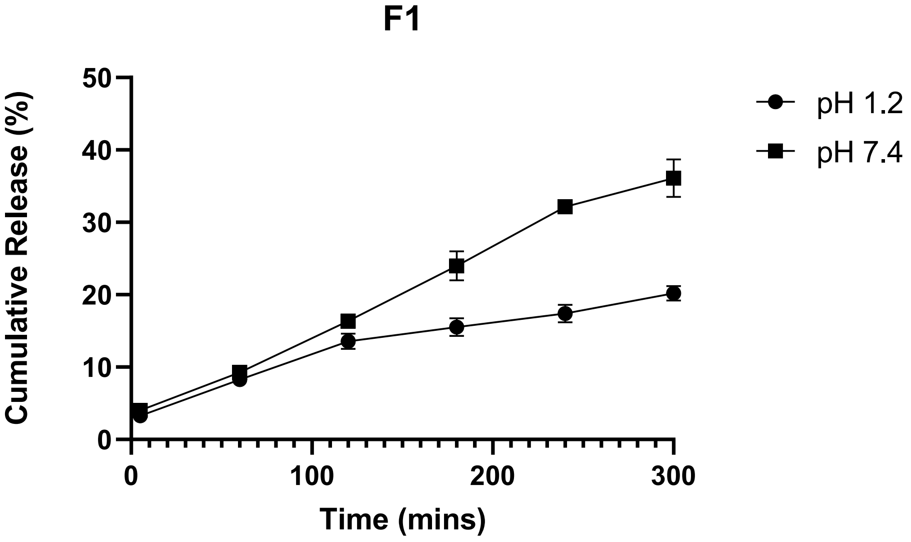

Figure S16: The release profile of F1 (mean ± SD, n=3) at each pH.

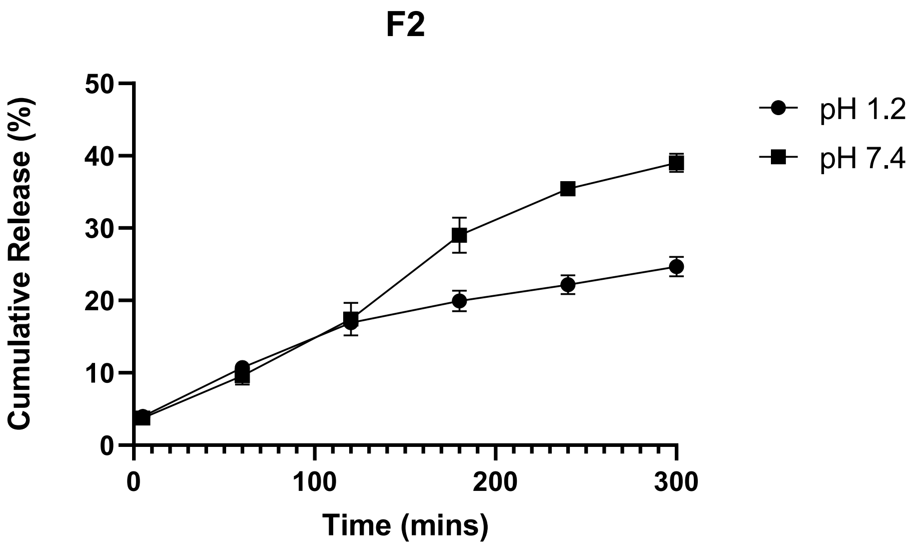

Figure S17: The release profile of F2 (mean ± SD, n=3) at each pH.

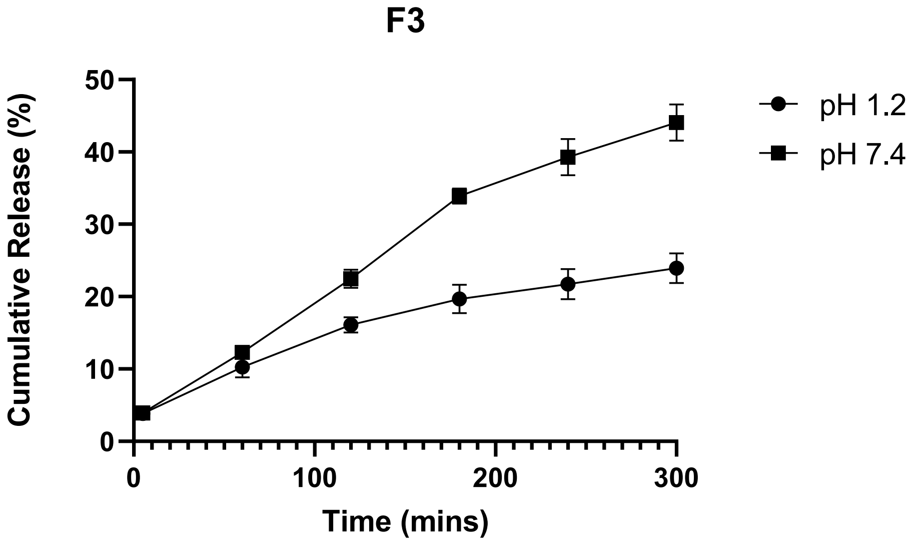

Figure S18: The release profile of F3 (mean ± SD, n=3) at each pH.

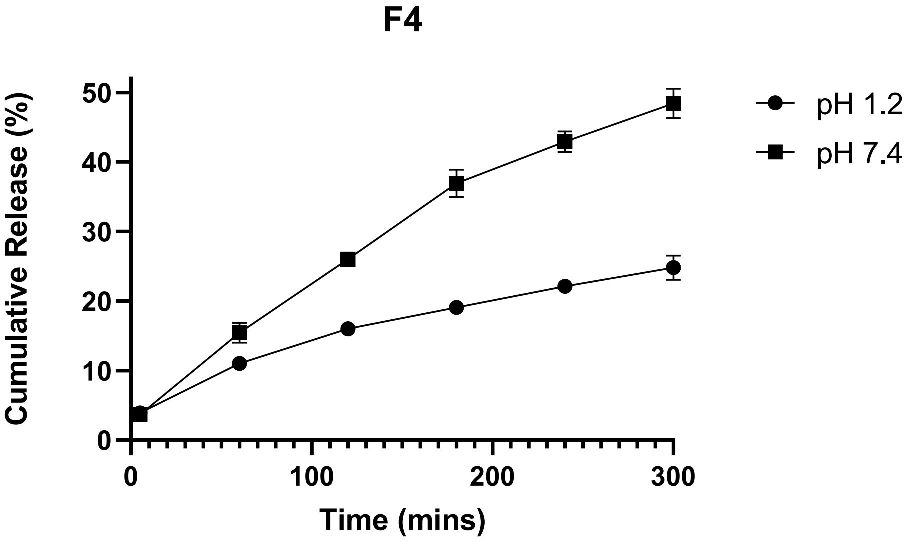

Figure S19: The release profile of F4 (mean ± SD, n=3) at each pH.

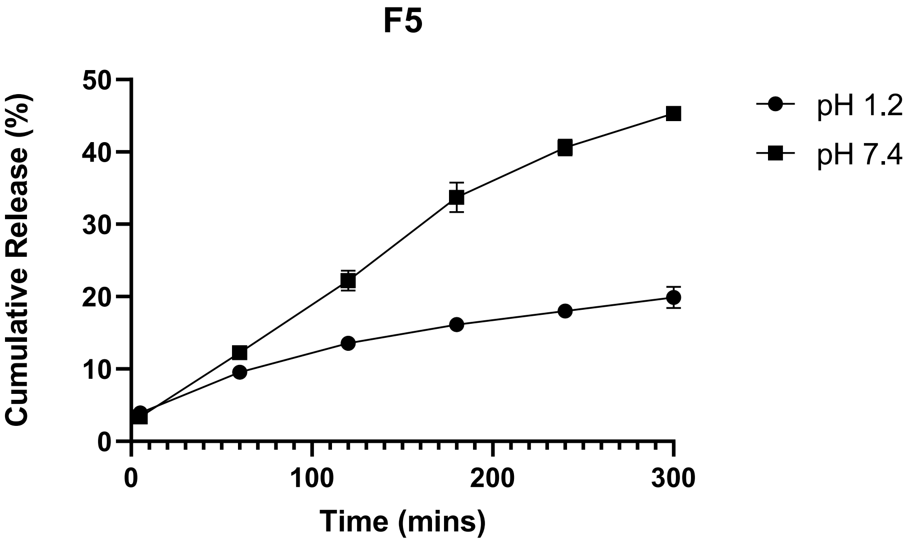

Figure S20: The release profile of F5 (mean ± SD, n=3) at each pH.

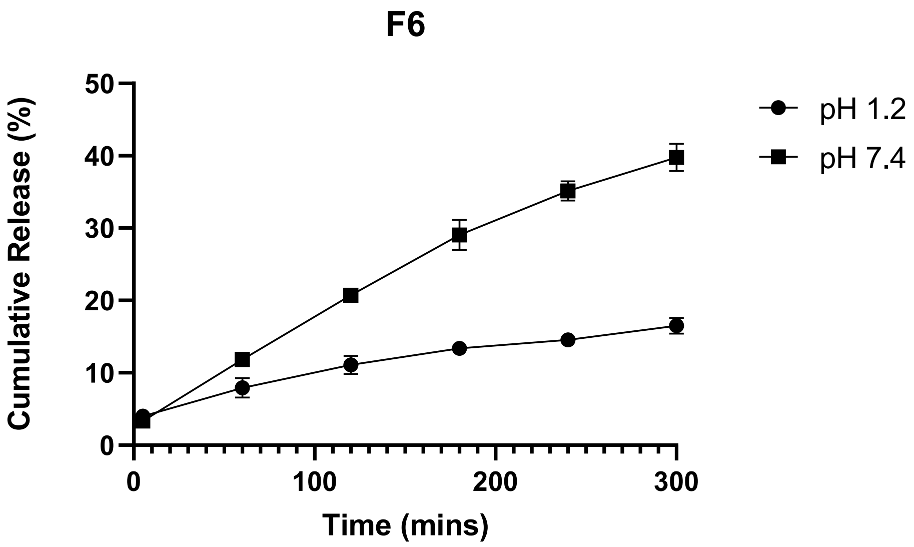

Figure S21: The release profile of F6 (mean ± SD, n=3) at each pH.

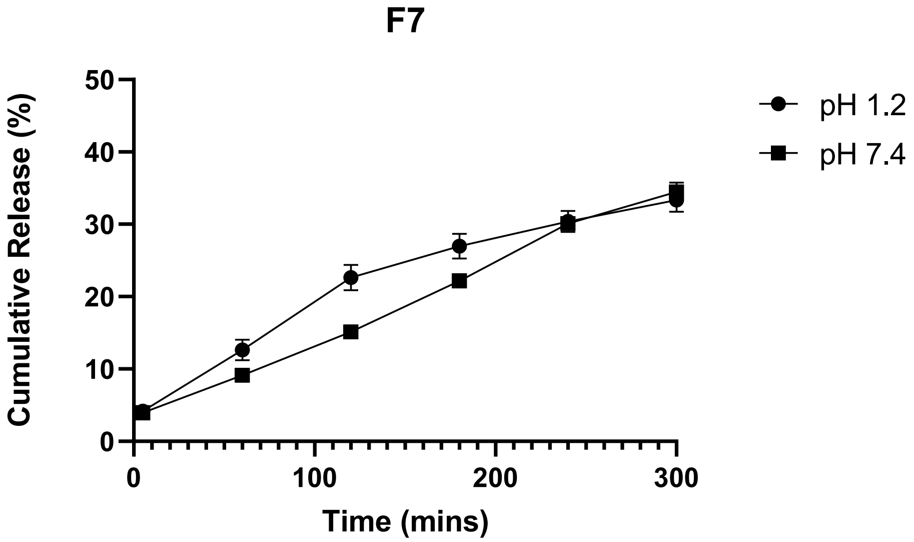

Figure S22: The release profile of F7 (mean ± SD, n=3) at each pH.

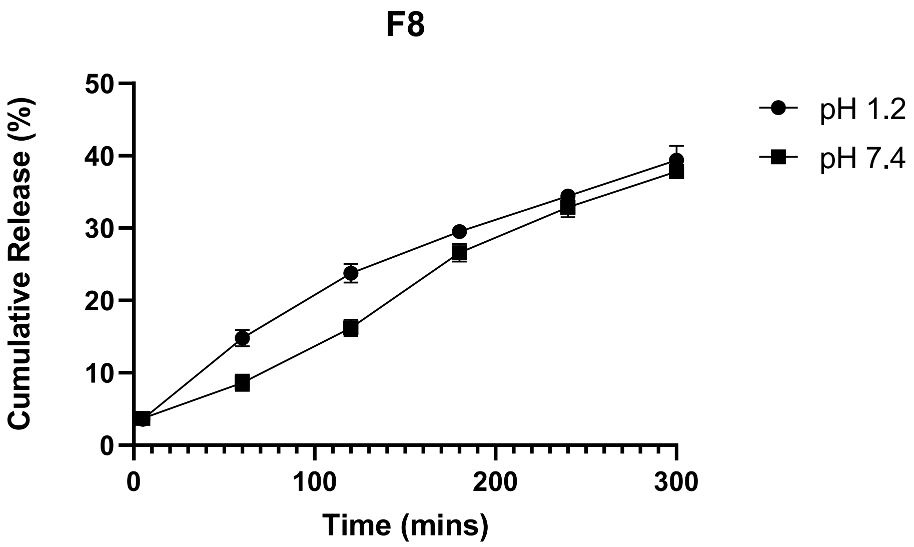

Figure S23: The release profile of F8 (mean ± SD, n=3) at each pH.

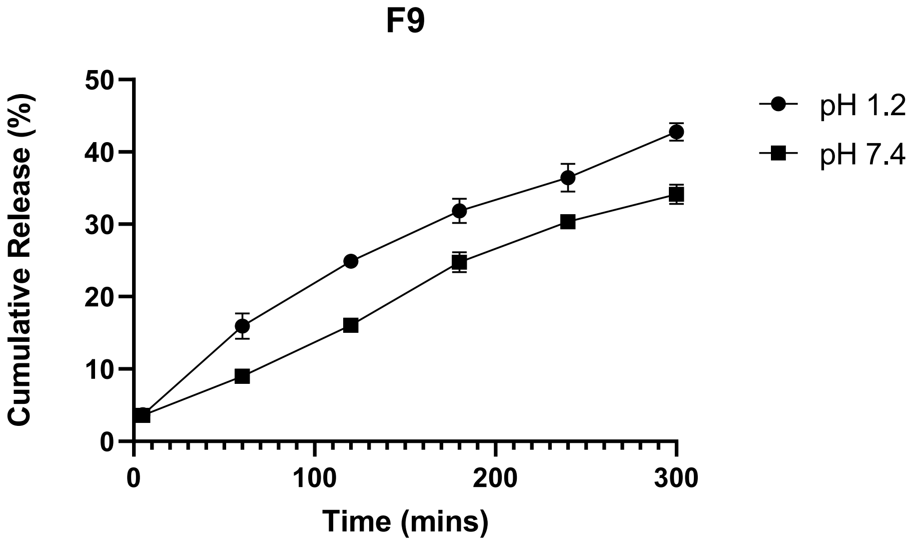

Figure S24: The release profile of F9 (mean ± SD, n=3) at each pH.
